# Overview of Thermal Management Solution for 3D Integrated Circuits Using Carbon-Nanotube-Based Silicon Through-Vias

**DOI:** 10.3390/mi16090968

**Published:** 2025-08-22

**Authors:** Heebo Ha, Hongju Kim, Sumin Lee, Sooyong Choi, Chunghyeon Choi, Wan Yusmawati Wan Yusoff, Ali Shan, Sooman Lim, Byungil Hwang

**Affiliations:** 1 Department of Intelligent Semiconductor Engineering, Chung-Ang University, Seoul 06974, Republic of Korea; hhb2340@naver.com (H.H.); dltnals9902@naver.com (S.L.); ovnd@cau.ac.kr (S.C.); chlcnd147@naver.com (C.C.); 2 School of Integrative Engineering, Chung-Ang University, Seoul 06974, Republic of Korea; a060074@cau.ac.kr; 3 Department of Physics, Centre for Defence Foundation Studies, Universiti Pertahanan Nasional Malaysia, Kem Sungai Besi, Kuala Lumpur 57000, Malaysia; yusmawati@upnm.edu.my; 4 Department of Flexible and Printable Electronics, LANL-JBNU Engineering Institute, Jeonbuk National University, Jeonju 54896, Republic of Korea; shan.ali139122@gmail.com

**Keywords:** CNT–Cu composites, thermal management, 3D integrated circuits (3D ICs), through-silicon vias (TSVs), carbon nanotubes (CNTs)

## Abstract

Three-dimensional integrated circuit (3D IC) technology is an innovative approach in the semiconductor industry aimed at enhancing performance and reducing power consumption. However, thermal management issues arising from high-density stacking pose significant challenges. Carbon nanotubes (CNTs) have gained attention as a promising material for addressing the thermal management problems of through-silicon vias (TSVs) owing to their unique properties, such as high thermal conductivity, electrical conductivity, excellent mechanical strength, and low coefficient of thermal expansion (CTE). This paper reviews various applications and the latest research results on CNT-based TSVs. Furthermore, it proposes a novel TSV design using CNT–copper–tin composites to optimize the performance and assess the feasibility of CNT-based TSVs.

## 1. Introduction

Three-dimensional integrated circuit (3D IC) technology has achieved groundbreaking advancements in the semiconductor industry in recent years and has drawn significant attention [1,2]. Three-dimensional ICs stack multiple semiconductor chips vertically, thereby maximizing spatial efficiency, shortening interchip connection distances, increasing data transfer speeds, and reducing power consumption [3,4,5]. This technology is essential in various applications, such as mobile devices, high-performance computing, artificial intelligence (AI), and the Internet of Things (IoT). Specifically, 3D ICs play a crucial role in applications requiring high performance and efficiency [6,7,8,9].

Three-dimensional IC chips offer several advantages. By reducing the connection distances between chips, 3D ICs minimize signal transmission delays, thereby significantly enhancing the overall system performance [10,11,12]. This is particularly advantageous in high-performance computing and data center applications, where high-speed data transmission is critical. Improved signal transmission speeds lead to faster data processing, which directly contributes to an overall improvement in system performance. Additionally, the shorter signal transmission distances in 3D ICs help to reduce power consumption [4]. This reduction is highly beneficial for mobile devices, where battery life is crucial, and for data centers, where energy efficiency is critical. Reduced power consumption increases the energy efficiency of the systems, thereby enabling environmentally sustainable designs. Finally, the vertical stacking of multiple chips in 3D ICs results in a much smaller size than that of 2D ICs with similar functionality [13]. This compact size offers significant advantages for applications with limited space, such as wearable devices and miniaturized sensor networks. A smaller package size increases design flexibility and enhances portability.

One of the core components of 3D ICs is the through-silicon via (TSV), which is responsible for vertical connections between chips [14,15,16]. TSVs penetrate silicon wafers to transmit electrical signals and power, thereby enabling efficient inter-chip communication. TSVs maximize system performance by supporting high-speed data transfer and power delivery. In addition to electrical connections, TSVs also act as thermal dissipation paths, helping to release the heat generated within the stacked chips to the outside, preventing overheating and ensuring stable operation [16,17,18,19]. However, the thermal management of high-density stacked structures remains a significant issue [20]. The first major challenge is thus thermal management. In a 3D IC structure, vertically stacked chips easily accumulate heat [20]. Although TSVs provide a pathway for heat dissipation, managing heat in high-density stacked structures remains problematic. Without proper heat dissipation mechanisms, the system performance can degrade, and, in severe cases, this can lead to system failure. Second, TSVs are exposed to various mechanical stresses [21,22]. During the stacking process, differences in thermal expansion can cause mechanical stress between the TSVs and the surrounding materials [23]. These stresses can compromise the structural integrity of the TSVs, thereby reducing their reliability. Thermal stress, particularly during thermal cycling, can shorten the lifespans of TSVs, as repeated expansion and contraction between copper and silicon create coefficient of thermal expansion (CTE) mismatch, leading to interfacial stress, microcrack initiation, and structural failure [24,25,26]. Finally, the electrical properties of TSVs vary significantly depending on the conductive materials and design used. Traditional copper-based TSVs offer excellent electrical performance but have limitations in thermal management [24]. New materials and designs that maintain electrical performance while addressing thermal management issues are required.

Carbon nanotubes (CNTs) are emerging as a promising material to overcome these challenges in TSV technology [27,28,29]. CNTs are nanometer-scale tubes composed of carbon atoms arranged in a hexagonal honeycomb structure that possesses unique physical and chemical properties. CNTs exhibit thermal conductivity exceeding 3000 W/mK, which is significantly higher than that of copper [30]. This allows efficient heat dissipation within the TSVs, effectively managing the excessive heat generated in high-density stacked structures [31]. In addition, CNTs possess metal-like electrical conductivity, which enables fast and efficient transmission of electrical signals [32,33]. This property helps the TSVs to maintain electrical connections while delivering signals at high speeds. CNTs also exhibit high tensile strength and flexibility, enhancing the structural reliability of TSVs, particularly in high-temperature and high-pressure environments [34,35]. The mechanical robustness of CNTs extends the lifespan and reliability of TSVs. Finally, CNTs have a near-zero CTE, which minimizes thermal stress during thermal cycling and maintains the structural integrity of TSVs across various temperature conditions [36].

Despite these promising properties, several challenges hinder the industrial application of CNT-based TSVs [37,38]. Achieving uniform CNT growth and vertical alignment using scalable processes such as PVD or PECVD presents a major challenge, as morphological changes can degrade thermal and electrical performance [39,40]. Interfacial bonding between CNTs and the surrounding materials is another critical issue, since weak adhesion can compromise mechanical reliability during thermal cycling [41,42,43]. To address these issues, CNT–Cu composite TSVs have been investigated, combining the high conductivity of copper with the stress-mitigating and structural robustness of CNTs; however, their integration still faces challenges in achieving uniform Cu filling and controlled CNT dispersion. In addition, scalable fabrication remains limited by the high cost of producing high-purity CNTs, process complexity, and compatibility with complementary metal–oxide–semiconductor (CMOS) back-end-of-line technologies. Long-term reliability under thermal and electrical stress also requires further validation, as microcrack initiation and electromigration have not yet been fully resolved. In addition, the lack of mass fabrication and testing standards limits comparability and delays industrial implementation.

**Figure 1:**
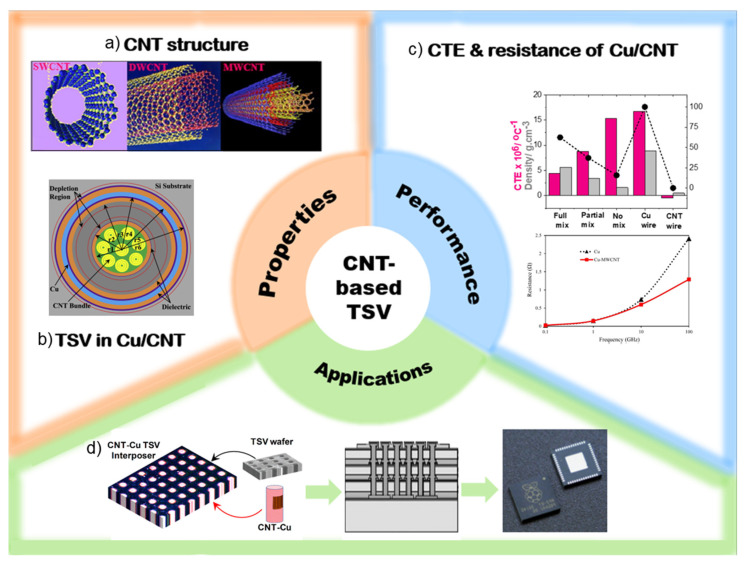
Overview of CNT-based through-silicon vias (TSVs): (**a**) representative CNT structures (SWCNT, DWCNT, and MWCNT), reprinted with permission from [44], copyright 2012 Elsevier; (**b**) schematic of a Cu/CNT-integrated TSV, reprinted with permission from [45], copyright 2023 Elsevier; (**c**) CTE and resistance in Cu/CNT composites, reprinted with permission from [46], copyright 2020 Elsevier; (**d**) application in 3D IC packaging using CNT–Cu interposers, reprinted with permission from [47], copyright 2018 American Chemical Society, [48] copyright 2010 Elsevier.

This review explores an innovative solution to the thermal management challenges in 3D integrated circuits (3D ICs) by utilizing carbon nanotube (CNT)-based TSVs. It aims to comprehensively review the related research, assess the potential of CNT-based TSVs, and provide insights into future research directions. By evaluating the thermal management performance of CNT-based TSVs and comparing it with that of traditional copper-based TSVs, this study aims to demonstrate their superiority. The remainder of this paper is organized as follows (Figure 1): The first section describes the properties of CNTs and discusses the design and fabrication methods of CNT-based TSVs. The second section evaluates the research on the thermal management performance, electrical performance, mechanical properties, reliability, and stability of CNT-based TSVs under various environmental conditions. The final section presents real-world application examples of CNT-based TSVs and suggests future research directions and potential advancements. This study contributes to the sustainable development of the semiconductor industry by introducing and proposing solutions to the thermal management issues of 3D ICs. Moreover, it aims to enhance the commercialization potential of CNT-based TSVs and promote the development of high-performance and highly reliable semiconductor devices.

## 2. Brief Overview of Properties of Carbon Nanotubes

**Figure 2:**
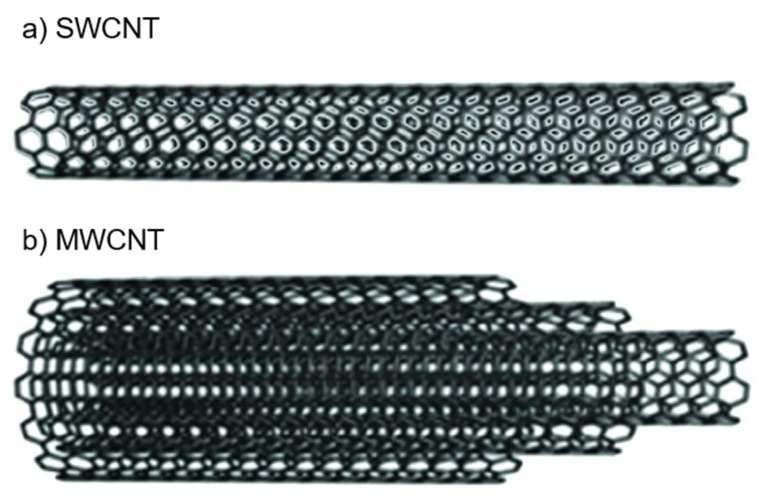
Structure of (**a**) single-walled and (**b**) multi-walled CNTs. Reprinted with permission from [51], copyright 2013 John Wiley and Sons.

Carbon nanotubes (CNTs) have gained attention in semiconductor technology and other applications owing to their unique physical and chemical properties. The characteristics of CNTs are primarily determined by their structure and synthesis method, and they exhibit excellent electrical, thermal, and mechanical properties [27,49,50]. CNTs are nanometer-sized tubes with carbon atoms arranged in a hexagonal honeycomb structure [51]. As shown in Figure 2, they are classified into single-walled carbon nanotubes (SWCNTs) and multi-walled carbon nanotubes (MWCNTs). SWCNTs consist of a single layer of graphene sheet rolled into a cylindrical shape, whereas MWCNTs consist of multiple graphene sheets concentrically layered [52]. This structure imparts unique physical and chemical properties to the CNTs, thereby offering diverse applications.

In addition, CNTs possess extremely high thermal conductivity, surpassing that of traditional thermally conductive materials such as copper [53]. For SWCNTs, the thermal conductivity reaches approximately 3500 W/mK, whereas, for MWCNTs, it is approximately 3000 W/mK [54]. This high thermal conductivity provides the potential to efficiently dissipate heat within TSVs. The thermal conductivity of CNTs can vary depending on their alignment, density, and length, with vertically aligned CNT structures maximizing heat transfer efficiency. This plays a crucial role in addressing the thermal management of TSVs. CNTs also exhibit metal-level electrical conductivity, contributing to the fast and efficient transmission of electrical signals [55,56]. The electrical conductivity of CNTs varies according to their structural characteristics, and SWCNTs exhibit both metallic and semiconducting properties. MWCNTs, owing to their multilayered structure, can further enhance electrical conductivity [49,57,58]. The high electrical conductivity of CNTs provides significant advantages for TSVs in terms of maintaining electrical connections and transmitting signals at high speeds. This is particularly important for modern electronic devices requiring high-speed data transmission and low power consumption. Furthermore, CNTs possess high tensile strength and flexibility, offering mechanical stability across various applications. The tensile strength of SWCNTs reaches approximately 100 GPa, far exceeding that of steel. MWCNTs also exhibit high mechanical strength and play a crucial role in maintaining structural integrity. These properties enhance the structural reliability of TSVs, ensuring their stability, particularly in high-temperature and high-pressure environments. The mechanical strength of CNTs is vital for TSVs to withstand thermal and mechanical stresses. In terms of thermal properties, CNTs have a near-zero CTE, which minimizes thermal stress due to temperature changes [36]. This characteristic is crucial for maintaining the structural integrity of TSVs at various temperatures. A low CTE extends the lifespan of TSVs during thermal cycling and enhances their reliability, particularly for applications involving significant temperature fluctuations [59]. CNTs exhibit excellent chemical stability and maintain stable performance under diverse environmental conditions. This means that CNTs are resistant to oxidation, corrosion, and other chemical reactions, ensuring that their performance does not degrade over long-term use. This property contributes to the high reliability of TSVs in various applications. In addition, CNTs remain stable at high temperatures, making them suitable for use in high-temperature processes. These unique properties of CNTs enhance their applicability not only in TSVs, but also across a range of electronic devices and systems. CNTs offer innovative solutions in areas such as high-speed data transmission, efficient power delivery, and thermal management. Specifically, CNT-based TSVs in 3D ICs have the potential to maximize the system performance, reduce power consumption, and effectively address thermal management issues. These properties position CNTs as key elements for the sustainable development of the semiconductor industry, and further research is likely to uncover more advanced applications.

## 3. Design and Fabrication of CNT-Based TSVs

CNT-based TSVs are designed to enhance their electrical and thermal performance. Typically, TSVs provide electrical connections by penetrating a silicon substrate [60]. Because of their high thermal and electrical conductivity, CNTs are ideal for maximizing the performance of TSVs. The arrangement and alignment of CNTs significantly affect the electrical and thermal performance of TSVs. Vertically aligned CNTs can maximize thermal conductivity, but the electrical conductivity can vary depending on their arrangement and alignment [56]. Optimizing the arrangement and alignment of CNTs to maximize their electrical conductivity is essential. This optimization can be achieved by precisely controlling the density, length, and alignment state of CNTs [61]. In particular, high-density vertically aligned CNT arrays can enhance both thermal and electrical conductivities.

Appropriate growth techniques are necessary for the application of CNTs to TSV structures. Chemical vapor deposition (CVD) is one of the most common methods for growing CNTs. In this method, carbon-source gases are introduced into a high-temperature reaction chamber, where CNTs grow on the catalyst surface. CVD has the advantage of controlling the alignment and density of CNTs [62]. In plasma-enhanced chemical vapor deposition (PECVD), plasma is used to facilitate the CNT growth reaction [63]. This method allows CNTs to grow at lower temperatures, making it suitable for use with temperature-sensitive substrates. PECVD enables the high-density growth of CNTs and can achieve aligned CNT arrays. Transition metal catalysts such as nickel (Ni), iron (Fe), and cobalt (Co) can be used during the growth process, where the size and distribution of the catalyst particles are crucial factors that determine the diameter and density of the CNTs [64]. Controlling the catalyst allows the growth of CNTs with the desired properties.

**Figure 3:**
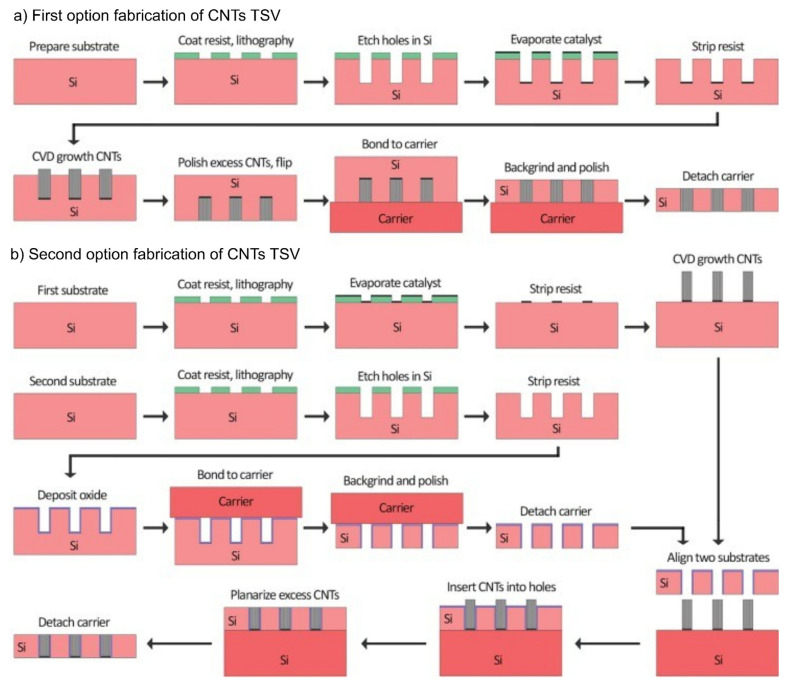
Options for the fabrication of CNT TSVs. (**a**) Direct growth of CNTs in blind silicon vias, followed by backgrinding and polishing. (**b**) Two-wafer method: CNTs are grown on a silicon substrate, inserted into pre-formed through-holes, and then backgrinded and polished. Reprinted with permission from [68], copyright 2024 Elsevier.

Two main methods exist for filling carbon nanotubes in TSVs: direct growth (bottom-up) and post-growth transfer (Figure 3) [65]. The direct growth method involves growing CNTs directly within the TSV, which is typically performed in high-temperature environments [66]. This method is suitable for small-diameter TSVs and can effectively grow CNTs with high conductivity and mechanical strength. First, the Fe and Al_2_O_3_ catalysts are deposited within the TSV structure. These catalysts act as key elements for CNT growth and are uniformly deposited on the TSV walls and bottom using physical deposition. Next, CNTs are grown using CVD. In this process, a carbon source gas (e.g., acetylene or ethylene) is decomposed at high temperatures to provide carbon atoms, forming CNTs on the catalyst surface [67]. The advantage of this method is that it allows uniform growth of high-density CNTs within the TSV, making it suitable for small-diameter TSVs and high-density 3D IC packaging. However, the high-temperature process step can make integration with other processes challenging, and maintaining the catalyst dispersion and activity during the growth process is crucial.

The post-growth transfer method involves transferring pregrown CNTs into TSVs, making it suitable for large TSV structures [69]. This method avoids high-temperature growth processes and increases process flexibility. First, CNTs are grown on a separate substrate. Subsequently, the inner walls and bottom of the TSV structure are prepared to facilitate the transfer of CNTs. The TSV walls are aligned to ensure proper insertion of the CNTs. Subsequently, the grown CNTs are transferred to the TSV structure. Precise positioning equipment and techniques are required to minimize alignment issues and ensure that the transferred CNTs are properly positioned and electrically connected within the TSV. The advantages of this method include performing the TSV transfer process at relatively low temperatures, even if CNT growth occurs at high temperatures, and its suitability for large TSV structures, benefiting high-density large-scale packaging. However, effective CNT transfer in small-diameter TSVs can be challenging because of alignment issues, and addressing damage or incomplete electrical connections during the transfer process is necessary. In both approaches, an additional step can be introduced to form composite TSVs, where copper is electroplated into the CNT-filled vias. This process infiltrates the porous CNT structures with copper, resulting in CNT–Cu hybrid interconnections that combine the high electrical conductivity of Cu with the superior thermal and mechanical properties of CNTs.

In conclusion, the direct growth (bottom-up) method is suitable for small-diameter TSVs and allows uniform CNT growth at high temperatures. By contrast, the post-growth transfer method is suitable for large TSVs but presents alignment challenges for small-diameter TSVs. Both methods require the optimization of the process conditions and the resolution of technical issues. Several issues may arise during the manufacturing of CNT-based TSVs, and various solutions are being researched to address them. The uniform growth of CNTs is a crucial factor in determining the performance of TSVs [37]. Therefore, optimizing the growth conditions and controlling the size and distribution of the catalyst particles are necessary. Additionally, high-precision growth techniques like PECVD can be used to ensure the uniformity of CNTs [63]. The electrical bonding between CNTs and TSVs significantly impacts the electrical performance of TSVs [70]. Techniques such as high-temperature annealing and electroplating can be used to strengthen the bonding areas [71]. Additionally, transition metal catalysts can improve the electrical conductivity of the bonding areas [72]. The mechanical stability of CNT-based TSVs can be affected by stress resulting from differences in thermal expansion [73]. To minimize this, it is necessary to optimize the arrangement and density of CNTs and reinforce the TSV structure. Moreover, the low CTE of CNTs can be utilized to minimize thermal stress. Finally, large-scale production of CNT-based TSVs remains a major technical challenge. To address this, high-efficiency growth techniques such as CVD and PECVD can be employed, and automated manufacturing processes can be introduced to increase production efficiency. In addition, enhancing quality control during production is crucial to ensure consistent product quality.

Following this, it is important to distinguish laboratory-scale from industrial approaches. Laboratory methods such as CVD and PECVD allow precise control over CNT growth, ensuring high alignment, density, and structural quality. However, these processes rely on highly purified carbon sources, expensive catalysts, and elevated growth temperatures, which limit throughput and increase production cost [74,75]. In contrast, industrial methods like batch processing or template-assisted electrodeposition are more suitable for large-scale manufacturing, but often suffer from non-uniform CNT length, partial misalignment, and weaker interfacial bonding, which can reduce device reliability. While single-walled CNTs are valuable due to their outstanding properties, they are still expensive and difficult to scale up, whereas multi-walled CNTs can be mass-produced but generally have greater quality variability, which significantly impacts their feasibility. Therefore, while small-scale experiments have clearly demonstrated the potential of CNT-based TSVs, practical applications depend on balancing material cost, scalability, and CMOS process compatibility.

## 4. Thermal Management Performance of CNT-Based TSVs

Turner’s model is commonly used to quantitatively estimate the effective CTE of CNT–Cu composites because it accounts for the mechanical interaction between constituent phases in a composite system. The model considers the influence of the CTE, bulk modulus, and volume fraction of each material, and is given by the following equation:

(1) $$\alpha_{composite}=\frac{\alpha_{Cu}\phi_{Cu}K_{Cu}+\alpha_{CNT}\phi_{CNT}K_{CNT}}{\phi_{Cu}K_{Cu}+\phi_{CNT}K_{CNT}}$$

Here, α represents the CTE, K is the bulk modulus, and ϕ denotes the volume fraction of each component. The bulk modulus K used in this equation can be calculated from the Young’s modulus (E) and Poisson’s ratio (ν), according to the following relationship:

(2) $$K=\frac{E}{3(1-2\nu )}$$

For example, the mechanical properties of MWCNTs vary significantly depending on the synthesis temperature. MWCNTs synthesized at 1200 °C exhibit a Young’s modulus of approximately 500 GPa and a Poisson’s ratio of 0.27. At 2600 °C, the Young’s modulus increases to 800 GPa, whereas the Poisson’s ratio remains the same. In comparison, copper exhibits a Young’s modulus of 109 GPa and a Poisson’s ratio of 0.34. Using these values, the bulk modulus of each material can be calculated and then applied to Turner’s model.

These theoretical predictions are further supported by numerous experimental studies that demonstrate the thermal management capabilities of CNT-based TSVs [76]. In experiments conducted by Chen et al., a CNT–Cu composite interposer was fabricated by synthesizing vertically aligned SWCNT pillars on a silicon substrate [47]. The CNT pillars were approximately 130 μm in diameter and 300 μm in height, characterized by high aspect ratios and a porosity of 95–98%. This high porosity facilitated the subsequent copper electrodeposition processes. To enhance the structural integrity and prevent collapse during electrodeposition, a controlled carbon-coating process was applied. Approximately 20% of the carbon was deposited in the CNT forest using CVD, effectively bonding the CNTs together and reinforcing the structure.

**Figure 4:**
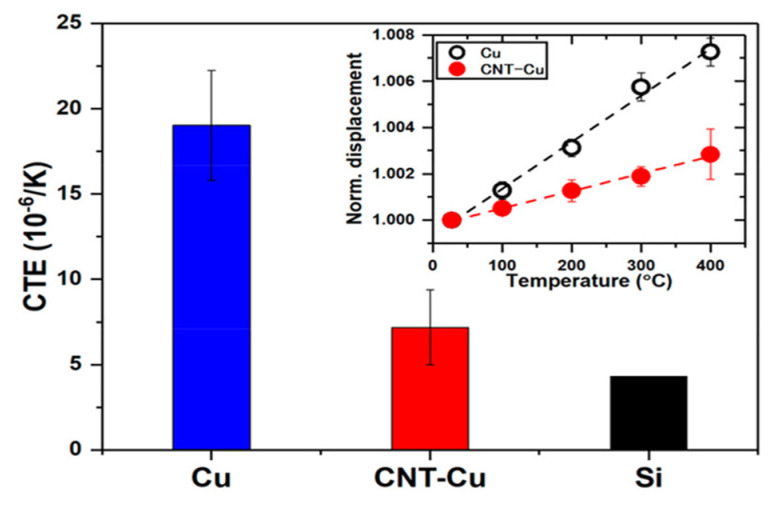
Coefficient of thermal expansion (CTE) comparison of the CNT–Cu composite pillar and Cu. The inset shows the temperature–expansion curve. Reprinted with permission from [47], copyright 2018 American Chemical Society.

The CTE of the CNT–Cu pillars was measured by analyzing their length expansion as a function of temperature (Figure 4). The results indicated that the CNT–Cu composite exhibited a CTE of approximately $7\times 10^{-6}/K$, which is significantly lower than that of pure copper ($19\times 10^{-6}/K$) and closer to that of silicon ($4.3\times 10^{-6}/K$). The reduced CTE mismatch between the CNT–Cu composite and silicon minimized the thermal stress at the interface, enhancing the reliability and lifespan of the TSV interposer. Ladani et al. also analyzed the dependence of the CTE and stress distribution of hybrid TSVs on the volume ratio of CNTs [77]. According to the study, as the volume ratio of CNTs increased, the CTE of the TSV structure gradually approached that of silicon (2.33 × $10^{-6}/K$). In particular, when the TSV was entirely composed of CNTs, the CTE decreased to a level almost identical to that of silicon. These findings indicate that, compared with TSVs composed solely of copper (14.3 × $10^{-6}/K$), the hybrid structure can significantly reduce thermal expansion mismatches and enhance thermomechanical reliability.

In this analysis, the CTE was evaluated in both longitudinal and transverse directions (Table 1). The longitudinal direction corresponds to the TSV axis, which penetrates the silicon substrate vertically, whereas the transverse direction refers to the horizontal plane perpendicular to that axis. This distinction is important because CNTs exhibit strong anisotropic properties owing to their alignment. Because CNTs are typically aligned along the longitudinal direction when grown inside TSVs, the reduction in CTE is more pronounced in that direction. As a result, the longitudinal CTE of the CNT-filled TSVs aligns more closely with that of silicon, whereas the transverse CTE remains slightly higher, owing to weaker alignment effects. This directional behavior further demonstrates the effectiveness of CNTs in alleviating thermal-mismatch-induced stresses along the TSV axis.

In addition, the incorporation of CNTs helped to redistribute stress concentrations, leading to a significant enhancement in the fatigue life of the solder joints, extending it by approximately three to four times during thermomechanical cycles. This improvement is attributed to the redistribution of stress from the copper–silicon interface to the CNT interior, which significantly reduces the likelihood of interface failure. These results highlight that the design of hybrid TSVs offers superior durability and reliability compared with those of conventional TSVs made solely of copper. Subramaniam et al. also reported that a CNT–Cu composite exhibited anisotropic thermal expansion behavior depending on the direction. The CTE was experimentally measured in both the axial direction (along the alignment of the CNTs) and the radial direction (perpendicular to the alignment). Within the temperature range of 250 K to 320 K, the axial CTE ranged from approximately 6.5 × 10^−6^/K to 5.6 × 10^−6^/K, whereas the radial CTE ranged from approximately 4.6 × 10^−6^/K to 3.6 × 10^−6^/K, indicating a lower expansion in the radial direction. This difference is attributed to the directional alignment of the CNTs within the copper matrix. Because CNTs inherently possess very low or even negative thermal expansion, their alignment plays a crucial role in suppressing the thermal strain. In the radial direction, where expansion acts against the cross section of the aligned CNTs, the structural rigidity of CNTs resists deformation more effectively, resulting in reduced thermal expansion. By contrast, in the axial direction, CNTs are aligned parallel to the expansion path, allowing slightly more elongation and, therefore, a higher CTE. This anisotropic behavior further demonstrates the capability of CNT–Cu composites to closely match the CTE of silicon, thereby enhancing thermomechanical compatibility for advanced electronic packaging applications.

Besides CNT–Cu TSVs, Cu TSVs offer excellent electrical conductivity but suffer from large CTE mismatch with silicon and significant electromigration issues, which limit their thermomechanical reliability under repeated thermal cycling [78]. Silicon-filled TSVs provide better CTE compatibility but exhibit relatively poor electrical and thermal conductivities, making them unsuitable for high-speed and high-power applications [79]. More recently, graphene-based TSVs have been investigated, owing to their outstanding electrical mobility and moderate thermal conductivity [80,81]. However, scalable integration of high-quality graphene into TSV structures remains a major challenge. In contrast, CNT-based TSVs uniquely combine metal-like electrical conductivity, ultra-high thermal conductivity, mechanical resilience, and low CTE, resulting in superior performance in both signal integrity and thermal stress management.

## 5. Performance, Reliability, and Fabrication Issues of CNT–CuComposite TSVs

**Figure 5:**
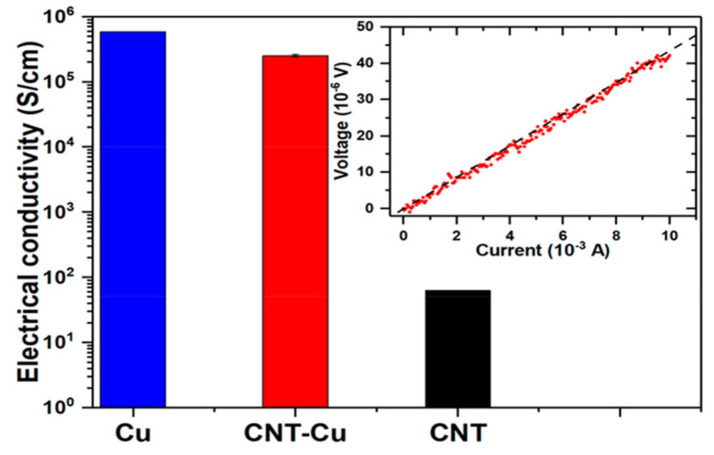
Electrical conductivity comparison between CNT–Cu composite pillar and Cu. The inset shows the I–V curve. Reprinted with permission from [47], copyright 2018 American Chemical Society.

In CNT–Cu composite TSVs, CNTs possess a CTE similar to that of silicon, effectively minimizing the interfacial thermal stress and thereby enhancing the mechanical reliability [82]. Furthermore, under thermal cycling conditions, the low CTE of CNTs helps to alleviate thermal stress, enabling the composite to maintain structural stability even under harsh thermal environments, such as high temperature and pressure [83]. In addition to these mechanical advantages, the CNT–Cu composite also demonstrates excellent electrical conductivity (Figure 5). Measured using the four-probe method, the conductivity reached approximately 2.5 × 10^5^ S/cm, which corresponds to approximately 43% of that of pure copper (5.8 × 10^5^ S/cm) [47]. This value is significantly higher than that of the pristine CNTs (~1 × 10^2^ S/cm), indicating the formation of efficient electrical pathways within the composite. The combination of high electrical conductivity and low CTE suggests that CNT–Cu composites offer a highly effective solution for electrical interconnection and thermal management in microelectronic packaging.

In terms of signal integrity, the inherent electrical properties of CNTs contribute to a reduced signal distortion and improved transmission speed [84]. The experimental results confirm that CNT–Cu TSVs maintain signal integrity even at high frequencies, providing significant advantages in high-speed data transmission applications such as 3D ICs. Moreover, from a power consumption perspective, CNT–Cu TSVs demonstrate a performance comparable to that of pure copper TSVs [85]. The improved thermal management leads to reduced power loss, thereby contributing to enhanced energy efficiency [86]. In conclusion, owing to their high electrical conductivity, superior mechanical strength, thermal stability, and energy efficiency, CNT–Cu composite TSVs are highly promising candidates for next-generation 3D IC interconnection applications.

Despite these promising attributes, CNT–Cu composites still face several critical challenges that hinder their widespread adoption in practical applications. One of the foremost issues is the weak interfacial interaction between the CNTs and the copper matrix. Owing to the poor wettability and limited chemical affinity between the CNTs and Cu, the resulting interfacial bonding is often insufficient, leading to increased contact resistance and compromised electron and phonon transport across the interface. This issue becomes even more pronounced when CNTs are not well dispersed, leading to agglomeration and the formation of voids, which further degrade the electrical, thermal, and mechanical properties. Another significant limitation is the difficulty of controlling the structural attributes of CNTs, such as their length, diameter, chirality, and alignment. These factors critically influence the composite performance; however, current CNT synthesis and integration techniques often produce heterogeneous CNT populations that are not easily scalable or uniform. Furthermore, during composite fabrication processes, especially powder-based mixing, CNTs are prone to damage, misalignment, or shortening, which negatively affect the final properties of the material. From a manufacturing perspective, the lack of mature, cost-effective, and scalable fabrication methods remains a major barrier. Techniques such as template-assisted electrodeposition offer improved CNT–Cu alignment and performance, but they are typically expensive and incompatible with mass production. Additionally, quality assurance and metrology standards specific to CNT–Cu composites are yet to be fully established, limiting their integration into existing semiconductor manufacturing lines. Compared with conventional copper TSVs, which provide good electrical conductivity but suffer from significant thermal stress due to CTE mismatch, CNT-filled TSVs exhibit intrinsically superior thermal conductivity (~3000–3500 W/mK) and exceptional tensile strength. However, the weak interfacial bonding between CNTs and the surrounding materials can compromise mechanical reliability. Hybrid CNT–Cu composites address this trade-off by combining the high conductivity of copper with the structural robustness and stress-mitigating properties of CNTs. Experimental studies have reported that CNT–Cu composites achieve thermal conductivities in the range of 600–1000 W/mK, surpassing those of pure copper (~400 W/mK), while simultaneously reducing interfacial stress mismatch with silicon [30,87,88]. In terms of mechanical reliability, CNT–Cu composites redistribute stress concentrations more effectively than either Cu or CNT alone, thereby offering enhanced bonding strength and fatigue resistance during thermal cycling. Therefore, ongoing research must address these key limitations through advances in improving CNT–metal bonding, achieving uniform scalable fabrication, and the standardization of testing.

## 6. Conclusions

This review explores the potential of CNT-based TSVs as a forward-looking strategy to address the thermal and structural challenges in 3D ICs. CNTs offer a unique combination of properties that align well with the complex demands of next-generation semiconductor packaging, owing to their remarkable thermal conductivity, electrical characteristics, mechanical resilience, and low thermal expansion. CNT–Cu composite TSVs have been highlighted as promising alternatives to conventional copper-based TSVs, particularly in applications requiring enhanced heat dissipation, dimensional stability, and reliable electrical interconnections under varying thermal and mechanical conditions. The intrinsic compatibility of CNTs with silicon in terms of thermal expansion helps to reduce interfacial stress, whereas their high conductivity supports signal integrity and system efficiency in densely stacked architectures. Despite these advantages, several key hurdles remain. Limited interfacial bonding between CNTs and the metal matrix, the aggregation of nanotubes, inconsistencies in the CNT structure, and challenges related to scalable, cost-efficient fabrication processes currently restrict their broader implementation. Furthermore, the lack of standardized processing techniques and evaluation protocols continues to be a barrier to their widespread industrial adoption. To address these challenges, researchers have been actively developing several approaches. Advanced catalyst engineering, low-temperature synthesis methods, and the optimization of CVD/PECVD parameters are being studied to improve CNT alignment and uniformity [89,90]. In addition, surface functionalization and interface engineering, such as metal nanoparticle decoration and chemical modification, are being employed to enhance CNT–metal bonding and reduce interfacial resistance [91,92]. These approaches provide future directions for technology-based research toward scalable manufacturing and industrial implementation. Future research should aim to refine the control of CNT dispersion and alignment, enhance CNT–metal interfacial engineering, and develop industry-compatible manufacturing approaches that ensure both performance and scalability. Establishing universal quality standards and metrology frameworks is critical for advancing commercialization. With continued interdisciplinary progress, CNT-based TSVs have the potential to become a central solution for enabling compact, high-performance, and thermally reliable 3D ICs for emerging technologies such as artificial intelligence, IoT, and advanced computing systems.

## Figures and Tables

**Table 1:** The CTE decreases with increasing CNT content in both the longitudinal and transverse directions [77].

CNT Content	$\mathrm{Longitudinal}\;\mathrm{CTE}\;(\times 10^{-6}/K)$	$\mathrm{Transverse}\;\mathrm{CTE}\;(\times 10^{-6}/K)$
0% (100% Cu)	4.00	3.90
25%	3.30	3.00
50%	2.80	2.50
75%	2.50	2.10
100% (All CNT)	2.30	1.80

## Data Availability

Not applicable.
